# Co-administration of drugs with parenteral nutrition in the neonatal intensive care unit—physical compatibility between three components

**DOI:** 10.1007/s00431-022-04466-z

**Published:** 2022-04-14

**Authors:** Niklas Nilsson, Ingebjørg Storesund, Ingunn Tho, Katerina Nezvalova-Henriksen

**Affiliations:** 1 Oslo University Hospital and Oslo Hospital Pharmacy, Hospital Pharmacies Enterprise, South-Eastern Norway, Oslo, Norway; 2 Department of Pharmacy, University of Oslo, Oslo, Norway; 3 Western Norway Hospital Pharmacy, Stavanger, Rogaland Norway; 4 Department of Clinical and Molecular Medicine, Norway University of Science and Technology, Trondheim, Norway

**Keywords:** Numeta G13E, Morphine, Dopamine, Cefotaxime, Precipitation, Oil-droplet growth

## Abstract

There is a lack of compatibility data for intravenous therapy to neonatal intensive care unit (NICU) patients, and the purpose of this study was to contribute with documented physical compatibility data to ensure safe co-administration. We selected Numeta G13E, the 3-in-1 parenteral nutrition (PN) used at our NICU, together with the frequently used drugs morphine, dopamine and cefotaxime in two- but also three-component combinations. Incompatibility may lead to particle formation (precipitation) and oil-droplet growth (emulsion destabilisation), both which are undesirable and pose a safety risk to already unstable patients. We assessed potential particle formation of three mixing ratios for each combination (always including 1 + 1 ratio) using light obscuration, turbidity and pH measurements combined with visual inspection by focused Tyndall beam. Potential droplet-growth and emulsion destabilisation was assessed by estimating PFAT5 from droplet size measurements and counts, mean droplet diameter and polydispersity index from dynamic light scattering, and pH measurements. Mixed samples were always compared to unmixed controls to capture changes as a result of mixing and samples were analysed directly after mixing and after 4 h to simulate long contact time. None of the samples showed any sign of precipitation, neither in the drug-drug nor in the two- or three-component mixture with PN. Neither did we detect any form of emulsion destabilisation.

*Conclusion*: Dopamine, morphine and cefotaxime were found to be compatible with NumetaG13E, and it is safe to co-administer these drugs together with this PN in NICU patients.

**Table:** 

**What is Known:** *• The need for co-administration of drugs and complex PN admixtures occurs frequently in NICU due to limited venous access.* *• Available compatibility data are scarce and for combinations of more than two components non-existent.*	
**What is New:** *• Here we report physical compatibility data of two- as well as three-component combinations of frequently used NICU drugs and a 3-in-1 PN admixture.* *• Co-administration of Numeta G13E with dopamine and morphine, but also with morphine and cefotaxime is safe in NICU.*

**What is Known:**

*• The need for co-administration of drugs and complex PN admixtures occurs frequently in NICU due to limited venous access.*

*• Available compatibility data are scarce and for combinations of more than two components non-existent.*

**What is New:**

*• Here we report physical compatibility data of two- as well as three-component combinations of frequently used NICU drugs and a 3-in-1 PN admixture.*

*• Co-administration of Numeta G13E with dopamine and morphine, but also with morphine and cefotaxime is safe in NICU.*

## Introduction

Preterm neonates hospitalised at the neonatal intensive care unit (NICU) need intravenous drug therapy and parenteral nutrition (PN) to ensure survival and proper growth and development. Due to their small size and narrow veins, most preterm neonates only tolerate the insertion of a single or double lumen central venous catheter (CVC) or peripherally inserted central catheter (PICC). Even though, the vascular access technology and culture has evolved, and jugular and brachiocephalic short and large catheters can be inserted under ultrasound guidance [1–3]. Limited venous access is a considerable challenge for the involved health care professionals when they have to administer several drugs, PN and blood transfusions intravenously and often together via the same catheter lumen [4]. Co-administration increases the risk of incompatibility reactions between the infused solutions because of differences in their physicochemical properties, composition and the complex nature of PN [4]. Consequences of incompatibilities between drugs and PN may result in formation of solid particles (precipitation) and oil-droplet expansion (lipid emulsion destabilisation). Resultant lumen occlusion, organ malfunction, oxidative stress and embolus formation have been reported [5–7]. Co-administration is off-label administration, as this practice is almost never described in the summary of product characteristics (SmPCs). This is in addition to the fact that most of the drugs used in NICU are off-label or unlicensed for other reasons [8]. Pausing infusions and flushing the intravenous lines prior to and after administration is a safety recommendation but might be undesirable in neonates due to hypervolemia and low fluid capacity. It is estimated that over 25% of co-administrations in NICU are incompatible and up to 75% are either incompatible or undocumented [4, 9].

Documented information about which drugs and PN may be compatible during Y-site administration is very scarce. There are a couple of retrospective studies reporting fatal embolism after infusion of incompatible drugs [10, 11]. However, compatibility studies cannot be performed in vivo due to ethical reasons; hence, there is a need for in vitro translational studies. Often even in vitro studies only describe combination of two components and the results are derived from analyses performed under predefined and not necessarily clinically relevant conditions [12]. Only a few original research studies on intravenous drug and PN compatibility in neonates have been published. Two studies analysed compatibilities with locally compounded PN, making their findings not generalisable [13, 14]. Nezvalova-Henriksen et al. found that paracetamol, vancomycin and fentanyl were all compatible with Numeta G13E at clinically relevant mixing ratios and infusion times [15]. Hammond et al. concluded that adrenaline, dobutamine, dopamine, morphine and milrinone were compatible with Plasma-Lyte 148 whereas furosemide and midazolam were not [16]. Staven et al. found that ampicillin, fosphenytoin and furosemide showed precipitation when mixed with Olimel N5E and Numeta G16E, whereas ceftazidime, clindamycin, dexamethasone, fluconazole, metronidazole, ondansetron and paracetamol were compatible [17, 18].

The results from these studies, whilst contributing to the information pool, are neither exhaustive nor generalisable and none reports on intravenous compatibility between more than two components at a time. In addition, only Hammond et al. [16], Nezvalova-Henriksen et al. [15] and Staven et al. [19] performed a battery of compatibility tests that would ensure the reliability and reproducibility of their results.

Our aim was to analyse the Y-site compatibility of dopamine, morphine, cefotaxime and Numeta G13E in a two- and three-component system. To the best of our knowledge, no documented compatibility information is available for such co-administration.

## Materials and methods

### Test materials

Our Neonatal Intensive Care Unit at Oslo University Hospital (OUS) utilises Numeta G13E® (Baxter) when preterm infants need PN. It is a 3-in-1 chamber bag that requires the addition of water-soluble vitamins (Soluvit®, Fresenius Kabi), lipid-soluble vitamins (Vitalipid infant®, Fresenius Kabi) and trace elements (Peditrace®, Fresenius Kabi) to be deemed complete or total. The detailed composition of Numeta G13E and additives used in this study are identical to those used by Nezvalova-Henriksen et al. [15]. In order to test the stability of Numeta G13E in extreme scenarios, the maximum amount of all additives was added according to manufacturer guidelines.

The test drugs, dopamine, morphine and cefotaxime, were selected based on the frequency of use at our NICU. An overview of dopamine, morphine and cefotaxime formulations, their dilution media and concentrations are presented in Table 1.

**Table 1:** Overview over drug formulations, excipients, dilution media and concentrations

**Drug**	**Excipients**	**Dilution medium**	**Final concentration**
Dopamine hydrochloride (Takeda) pH: 2.5–4.5 Lot.nr: 11,512,398	Sodium pyrosulphate, sodium chloride, water for injection	Undiluted	2 mg/ml
Morphine hydrochloride (Orion) pH: 3.0–5.0 Lot.nr: 41,210,619	Sodium chloride, hydrochloric acid, water for injection	Glucose 50 mg/ml	0.2 mg/ml
Cefotaxime (Villerton and MIP Pharma) pH: 5.0–7.5 (after dilution) Lot.nr: GNC2039	–	Glucose 50 mg/ml	40 mg/ml

### Study design

To replicate potential mixing ratios between the selected drugs and PN in the catheter, infusion rates were utilised as described by Nezvalova-Henriksen et al. [15]. The amount of PN was based on ESPGHAN nutrition requirements for neonatal and paediatric patients, and the estimates covered bodyweights from 0.5 to 10 kg [20]. Drug doses used to calculate infusion rates were based on national neonatal therapy guidelines and local syringe pump protocols as well as information from Kinderformularium [21]. Calculated mixing ratios selected for two- and three-component mixtures are presented in Table 2. Cefotaxime was mainly tested at high concentrations (40 mg/ml), but a lower concentration (10 mg/ml) was also evaluated in combination with morphine (1 + 1 and 30 + 1) and for the three-component mixture with morphine and PN (1 + 30 + 20, 1 + 9 + 1, 1 + 1 + 1). All mixing ratios are given in volumes of each component.

**Table 2:** Overview of two- and three-component mixtures and mixing ratio of drug + PN, drug + drug and drug + drug + PN

**Morphine + PN**	**Cefotaxime + PN**	**Dopamine + PN**	**Cefotaxime + morphine**	**Dopamine + morphine**	**Cefotaxime + morphine + PN**	**Dopamine + morphine + PN**
1 + 1	1 + 1	1 + 1	1 + 1	1 + 1	1 + 1 + 1	1 + 1 + 1
1 + 7	9 + 1	1 + 6	1 + 2	1 + 8	1 + 2 + 20	1 + 1 + 10
1 + 39	1 + 20	1 + 56	9 + 1	40 + 1	9 + 1 + 2	1 + 4 + 10
						4 + 1 + 10

### Preparation of samples

Because the lipid component of a 3-in-1 PN is an emulsion, which makes the admixture milky and opaque, the possibility to detect precipitation is lost. Therefore, the lipid compartment was replaced with Milli-Q water for studies of potential precipitation. This version of PN admixture will be referred to as *aq*Numeta G13E. No vitamins were added since water-soluble vitamins give a strong colour which might influence the analyses, and lipid-soluble vitamins make the solution cloudy or opaque. Only trace elements and electrolytes were added to *aq*Numeta G13E.

For the analysis of emulsion stability, all three chambers of Numeta G13E were mixed and maximum amounts of water-soluble vitamins (15 ml), lipid-soluble vitamins (25 ml), trace elements (15 ml), phosphate (2.5 mmol) and calcium gluconate (3.5 mmol) were added to the bag as specified by the manufacturer. This version will be referred to as Numeta G13E + .

All samples (i.e. mixing ratios of various volumes of drugs and/or PN) and controls were prepared at room temperature and filtered through a 0.22-µm syringe filter (VWR, Radnor, PA, USA), except for lipid containing admixtures. To check reproducibility, three replications of each mixing ratio of drug and PN were prepared and analysed, both for immediate and 4-h sample. Also, the unmixed controls were analysed in replications. All results are reported as mean and standard deviation (SD).

### Analyses

In order to assess the physical compatibility, a number of well-established analysis methods were used [22]. Since all analytical methods have their strengths and weaknesses, and incompatibility reactions can present themselves differently, conclusion regarding drug compatibility should not be drawn based on one method alone but be based on supportive information drawn from several methods. All samples were tested immediately after mixing and after 4 h. Controls of unmixed drugs and/or PN admixtures were measured in all analyses and compared to the mixed samples.

#### Methods for detection of particle precipitation

Samples of drug + drug and drug + *aq*Numeta G13E two- and three-component combinations were analysed for possible particle formation. Sub-visual particle counting was carried out by light obscuration (Accusizer Syringe Injection Sampler, Optical Particle Sizer, PSSNICOMP, Billerica, MA, USA) to estimate the total number of particles/ml of sizes ≥ 0.5 µm, 5 µm, 10 µm and 25 µm, respectively. The acceptance criteria were not more than a total of 2000 particles/ml ≥ 0.5 µm [22], whilst larger particles were not to exceed the limits for “large volume parenterals” of the Pharmacopoeia (not more than 25 particles/ml ≥ 10 µm or not more than 3 particles/ml ≥ 25 µm) [23]. The total number of particles ≥ 5 µm was included because particles in this size range could potentially block capillaries. A limit of not more than 100 particles/ml ≥ 0.5 µm was employed as acceptable background of particles in Milli-Q water and sampling tubes.

Turbidity measurements (2100Qis Turbidimeter, Hach Lange GmbH, Duesseldorf, Germany) required samples to not exceed 0.2–0.3 formazine nephelometry units (FNU) higher than the unmixed control FNU values [22].

Visual examination was used to detect precipitation or colour changes utilising two different light sources. The sample, in flat-bottom tubes, was placed above a fiberoptic Tyndall beam (Schott KL 1600 LED, Germany) and inspected. The sample was also inspected with a red laser pen (630–650 nm, P 3010 RoHS, Chongqing, China) shining perpendicularly through it. A Tyndall effect (i.e. visible red line throughout the sample) was interpreted as identification of particles, even though particles could not be seen with the naked eye. Both analyses were carried out in a dark room against a black background [24].

pH measurements were carried out using a pH metre (Seven Compact, Mettler Toledo, Greifensee, Switzerland). A change of > 1.0 pH unit for mixed samples as compared with the unmixed controls was seen as alarming, and depending on the solubility of the drug, was considered to potentially induce precipitation. For samples with PN, a pH > approximately 7.2 was regarded as alarming, since this could induce the risk of forming poorly soluble calcium phosphate precipitate [25].

#### Methods for analysing emulsion stability

Two- and three-component mixtures of drug + Numeta G13E + were investigated. Initial signs of destabilisation of an emulsion can be seen as a growth in oil-droplet size detected in the large diameter tail of the droplet size distribution. This was evaluated by droplet counting using light obscuration in extinction mode (Accusizer Syringe Injection Sampler, Optical Particle Sizer, PSS NICOMP, Billerica, MA, USA) and calculating the fraction of the large diameter oil-droplets (PFAT5: percentage of fat residing in globules larger than 5 µm). For details regarding preparation, instrument settings and calculation of PFAT5, please refer to previous papers [15, 18].

Later in the destabilisation process, the mean hydrodynamic diameter of the oil-droplet and polydispersity index (PDI) of the droplet size distribution will increase; therefore, these parameters were measured using dynamic light scattering (Zetasizer nano series, Malvern instruments, Malvern, UK). The Z-average mean size was used as a mean droplet diameter (MDD). According to USP, MDD of injectable emulsions should be < 500 nm [26]. A PDI below 0.2 was regarded as a monodisperse size distribution and hence a stable sample.

Again, pH of the mixed samples was compared to the unmixed controls. pH values below 5.5 reduce droplet repulsion forces and increase the probability of droplet coalescence and thereby emulsion destabilisation [27].

#### Statistical evaluation

Average and SD were calculated for all results. Compatibility was evaluated based on the overall results from several methods including stated acceptance criteria and controls combined with theoretical assessments based on pH and physico-chemical properties of drugs and TPN. An overall assessment of these factors was considered more appropriate than isolated statistical analysis.

## Results

### Analyses of potential particle precipitation

In all controls, samples with drug + drug combinations and drug(s) + *aq*Numeta G13E + combinations, both two- or three-component, low sub-visual particle counts were seen immediately after mixing and also after 4 h (Table 3). In the three-component mixture of cefotaxime (40 mg/ml), morphine and *aq*Numeta G13E + , the total sub-visual particle count was slightly increased for all mixing ratios yet well within the acceptance criteria of 2000 particle/ml > 0.5 µm. Importantly, larger particle counts (> 5, 10 and 25 µm) were also well within the limits (data not shown). Of note is that the controls from the same test set also had relatively high sub-visual particle counts.

**Table 3:** Results from precipitation testing after mixing cefotaxime 40 mg/ml, dopamine 2 mg/ml, morphine 0.2 mg/ml and aqNumeta G13E + in different mixing ratios (bold font indicates values outside acceptance criteria) (average ± SD; *n* = 3)

**Drug**		**Mix ratio**	**Particles/ml ≥ 0.5 μm**			**Turbidity (FNU)**		**pH**	
			0 h	4 h		0 h	4 h	0 h	4 h
aqNumeta G13E +		Control	149 ± 114	102 ± 61		0.17 ± 0.06	0.15 ± 0.05	5.71 ± 0.23	5.72 ± 0.20
Morphine		Control	124 ± 78	78 ± 21		0.13 ± 0.02	0.14 ± 0.01	4.55 ± 0.18	4.48 ± 0.09
Dopamine		Control	121 ± 32	69 ± 29		0.13 ± 0.01	0.14 ± 0.01	3.84 ± 0.08	3.87 ± 0.09
Cefotaxime		Control	65 ± 23	93 ± 11		0.17 ± 0.01	0.17 ± 0.01	5.40 ± 0.06	5.36 ± 0.06
Two-component analysis (drug + PN)	Morphine + aqNumeta G13E +	1 + 1	696 ± 92		358 ± 143^a^	0.14 ± 0.01	0.12 ± 0.01	5.92 ± 0.00	6.01 ± 0.04
		1 + 7	528 ± 183		243 ± 110	0.13 ± 0.02	0.12 ± 0.01	5.76 ± 0.07	5.69 ± 0.04
		1 + 39	248 ± 82		282 ± 55	0.13 ± 0.00	0.12 ± 0.02	5.84 ± 0.01	5.87 ± 0.02
	Cefotaxime + aqNumeta G13E +	1 + 1	135 ± 27		94 ± 9	0.30 ± 0.07	0.18 ± 0.02	5.79 ± 0.01	5.76 ± 0.01
		9 + 1	74 ± 25		152 ± 26	0.22 ± 0.02	0.17 ± 0.02	5.77 ± 0.03	5.66 ± 0.03
		1 + 20	191 ± 156		56 ± 13	0.15 ± 0.01	0.14 ± 0.04	5.86 ± 0.01	5.87 ± 0.01
	Dopamine + aqNumeta G13E +	1 + 1	585 ± 236		401 ± 94	0.14 ± 0.04	0.12 ± 0.01	5.67 ± 0.01	5.69 ± 0.01
		1 + 6	724 ± 228		400 ± 155	0.13 ± 0.02	0.13 ± 0.02	5.77 ± 0.02	5.78 ± 0.01
		1 + 56	434 ± 97		252 ± 90	0.13 ± 0.02	0.13 ± 0.02	5.81 ± 0.01	5.81 ± 0.01
Two-component analysis (drug + drug)	Cefotaxime + morphine	1 + 1	38 ± 18		51 ± 5	0.14 ± 0.03	0.13 ± 0.04	5.20 ± 0.02	5.16 ± 0.06
		1 + 2	42 ± 36		59 ± 50	0.18 ± 0.04	0.20 ± 0.02	4.88 ± 0.33	4.90 ± 0.20
		9 + 1	103 ± 32		190 ± 59	0.13 ± 0.03	0.14 ± 0.01	5.29 ± 0.01	5.07 ± 0.03
	Dopamine + morphine	1 + 1	59 ± 68		88 ± 35	0.13 ± 0.01	0.13 ± 0.01	4.08 ± 0.02	4.04 ± 0.01
		1 + 8	126 ± 29		68 ± 13	0.13 ± 0.01	0.13 ± 0.01	4.31 ± 0.01	4.29 ± 0.01
		40 + 1	58 ± 21		113 ± 65	0.14 ± 0.00	0.14 ± 0.00	3.82 ± 0.04	3.77 ± 0.04
Three-component analysis (drug + morphine + PN)	Dopamine + morphine + aqNumeta G13E +	1 + 1 + 1	10 ± 3		14 ± 2	0.13 ± 0.01	0.13 ± 0.03	5.87 ± 0.03	5.95 ± 0.01
		1 + 1 + 10	127 ± 51		102 ± 32	0.18 ± 0.03	0.17 ± 0.03	5.68 ± 0.02	5.66 ± 0.01
		1 + 4 + 10	172 ± 32		134 ± 62	0.15 ± 0.01	0.14 ± 0.01	5.85 ± 0.01	5.85 ± 0.01
		4 + 1 + 10	114 ± 63		77 ± 58	0.17 ± 0.03	0.16 ± 0.03	5.80 ± 0.01	5.80 ± 0.01
	Cefotaxime + morphine + aqNumeta G13E +	1 + 1 + 1	241 ± 143		440 ± 156	0.09 ± 0.02	0.11 ± 0.03	5.74 ± 0.01	5.71 ± 0.02
		1 + 2 + 20	362 ± 87		719 ± 282	0.16 ± 0.01	0.21 ± 0.07	5.81 ± 0.03	5.77 ± 0.01
		9 + 1 + 2	1120 ± 662		678 ± 183	0.13 ± 0.04	0.15 ± 0.03	5.61 ± 0.01	5.60 ± 0.00

^a^Result is based on two parallels

All controls and mixed samples showed low turbidity (Table 3). Slightly elevated turbidity in samples of cefotaxime with morphine were detected, but the values were within the acceptance criteria.

Upon visual inspection, none of the samples showed any signs of precipitation. However, *aq*Numeta G13E + itself (control) showed signs of a weak inherent Tyndall effect, which could also be seen in mixtures with the drugs. Reconstituted cefotaxime (control) had a weak yellow colour and gave rise to a weak Tyndall effect which could be traced to some samples when mixed with *aq*Numeta G13E + .

When it comes to pH, no alarming changes were seen for any of the mixed samples during the analysis time range of 4 h, and the pH values of the mixtures were found to mirror the unmixed controls (Table 3).

In addition to the main test design, cefotaxime was analysed using a lower drug concentration (10 mg/ml) in a two-component combination of cefotaxime with morphine and in a three-component combination with morphine and aqNumeta G13E + . All these samples were stable and within acceptance criteria in all analyses for both two- and three-component mixtures (data not shown).

### Analyses of potential emulsion destabilisation

PFAT5 values are presented in Table 4, and in most combinations PFAT5 was below the recommended limit for parenteral nutrition (PFAT5 < 0.4%) [27]. Only two mixing ratios showed slightly increased PFAT5 results but only slightly above the threshold. This was in a sample of dopamine and Numeta G13E + (1 + 56) at both time points and in a sample of cefotaxime and Numeta G13E + (1 + 1) after 4 h.

**Table 4:** Results from emulsion stability analyses when drug was mixed with Numeta G13E + (average ± SD; *n* = 3)

**Drug**		**Mix ratio**	**Z-average (nm)**	**PDI**	**%PFAT5**		**pH**	
					0 h	4 h	0 h	4 h
Numeta G13E +		Control	248 ± 2	0.13 ± 0.02	0.23	0.12	5.80 ± 0.07	5.79 ± 0.05
Two-component analysis (drug + PN)	Morphine + Numeta G13E +	1 + 1	249 ± 1	0.13 ± 0.02	0.19 ± 0.03	0.19 ± 0.01^a^	5.92 ± 0.01	5.90 ± 0.01
		1 + 7	249 ± 2	0.13 ± 0.01	0.29 ± 0.05	0.20 ± 0.05	5.81 ± 0.02	5.84 ± 0.03
		1 + 39	247 ± 2	0.15 ± 0.03	0.31 ± 0.08	0.25 ± 0.05	5.79 ± 0.01	5.79 ± 0.01
	Cefotaxime + Numeta G13E +	1 + 1	252 ± 2	0.13 ± 0.02	0.28 ± 0.04	0.41 ± 0.05^*^	5.71 ± 0.01	5.66 ± 0.02
		9 + 1	248 ± 2	0.14 ± 0.03	0.24 ± 0.09	0.15 ± 0.15	5.84 ± 0.01	5.86 ± 0.01
		1 + 20	249 ± 2	0.12 ± 0.01	0.39 ± 0.04	0.24 ± 0.02	5.84 ± 0.02	5.83 ± 0.02
	Dopamine + Numeta G13E +	1 + 1	248 ± 1	0.13 ± 0.02	0.24 ± 0.03	0.23 ± 0.05	5.86 ± 0.01	5.89 ± 0.01
		1 + 6	247 ± 2	0.15 ± 0.02	0.25 ± 0.02	0.22 ± 0.03	5.67 ± 0.01	5.66 ± 0.03
		1 + 56	248 ± 2	0.13 ± 0.02	0.42 ± 0.09^*^	0.46 ± 0.06^*^	5.81 ± 0.02	5.83 ± 0.03
Three-component analysis (drug + morphine + PN)	Dopamine + morphine + Numeta G13E +	1 + 1 + 1	248 ± 2	0.14 ± 0.02	0.05 ± 0.01	0.16 ± 0.03	5.90 ± 0.01	5.87 ± 0.01
		1 + 1 + 10	248 ± 3	0.13 ± 0.02	0.07 ± 0.01	0.16 ± 0.01	5.81 ± 0.01	5.81 ± 0.01
		1 + 4 + 10	249 ± 2	0.12 ± 0.01	0.12 ± 0.04	0.20 ± 0.08	5.82 ± 0.01	5.86 ± 0.01
		4 + 1 + 10	249 ± 3	0.12 ± 0.01	0.08 ± 0.03	0.19 ± 0.06	5.82 ± 0.03	5.80 ± 0.01
	Cefotaxime + morphine + Numeta G13E +	1 + 1 + 1	240 ± 2	0.09 ± 0.03	0.05 ± 0.01	0.03 ± 0.01	5.69 ± 0.02	5.66 ± 0.01
		1 + 2 + 20	272 ± 31	0.20 ± 0.07	0.19 ± 0.04	0.15 ± 0.06	5.81 ± 0.03	5.81 ± 0.03
		9 + 1 + 2	279 ± 2	0.12 ± 0.01	0.06 ± 0.01	0.03 ± 0.00	5.45 ± 0.02	5.44 ± 0.01

^a^One parallel/sample was contaminated and was excluded

^*^Values outside the acceptance criteria

All mixed combinations of two as well as three components showed low and stable mean droplet diameter in the range of 240 to 280 nm (Z-average) and small polydispersity indexes. The variations observed can be traced back to differences between the PN bags (batches) used in the test set. The pH values of mixed samples were similar to the unmixed control of Numeta G13E + (Table 4).

## Discussion

We can conclude that there were no signs of particle precipitation nor emulsion destabilisation in simulated co-administration of dopamine, morphine and cefotaxime with Numeta G13E, either in drug + drug combination or in a two- or three-component mixture with Numeta G13E in our study.

Most compatibility studies involving morphine have been done using morphine sulphate [28]. However, morphine products available in the local NICU (as in the rest of Scandinavia) are morphine hydrochloride. Morphine (Mw 285.3 g/mol) has a pKa of 8.21 [29], and the main difference between morphine sulphate (Mw 668.8 g/mol) and morphine hydrochloride (Mw 321.8 g/mol) is the different aqueous solubility (1:15.5 and 1:17.5, respectively). To the best of our knowledge, no other studies have investigated the compatibility of morphine hydrochloride with dopamine or cefotaxime nor with 3-in-1 PN admixtures. Trissel et al. studied the physical compatibility of morphine sulphate 15 mg/ml and 1 mg/ml, and found that the high concentration was incompatible with nine parenteral nutrition formulations (emulsion destabilisation) whereas morphine sulphate 1 mg/ml was compatible with all PN in their study [30]. As the current study addresses neonates, a clinically relevant morphine concentration of 0.2 mg/ml was used. The finding that low concentration of morphine hydrochloride is compatible with Numeta G13E supports the hypothesis that morphine could have a concentration dependent emulsion destabilisation effect [30]. When it comes to potential precipitation, Trissel et al. used a test setup where the lipid components were removed by centrifugation [30]. However, Staven et al. has shown that a similar setup left traces of lipids and surfactants in the aqueous phase which interfered with light obscuration and turbidity measurements [22]. Therefore, Staven’s and our assessments of potential precipitation were performed after substituting the liquid volume of the lipid phase with water. Neither Staven’s nor our study showed signs of precipitation.

Samples of dopamine 2 mg/ml mixed with *aq*Numeta G13E were compatible and showed low turbidity, low sub-visual particle count and stable pH. Trissel et al. on the other hand found dopamine 3.2 mg/ml to be incompatible with two of the central line PN formulations whereas seven other PN formulations were found compatible [30]. It is difficult to make direct comparisons since a different test setup was used. The three-component mixture of dopamine, morphine and *aq*Numeta G13E did not reveal any surprises after finding the two-component mixtures compatible; this was also compatible. When it comes to emulsion stability, there was one mixing ratio (1 + 56) of dopamine and Numeta G13E + that showed slightly elevated PFAT5 values. Strictly interpreted, this would be an indication of droplet growth and the beginning of emulsion destabilisation. However, the average values observed for these samples were very close to the acceptance limit of 0.4% suggested by Driscoll et al. [27]. Moreover, the three-component mixture of morphine, dopamine and Numeta G13E was found to be compatible in all mixing ratios, which suggests that the slight increase in PFAT5 in the one mixing ratio of the two-component combination could be a reversible aggregation of droplets rather than droplet coalescence [18]. Baptista et al. analysed emulsion stability by visual observation and did not see any disruption of the emulsion after mixing dopamine and PN [31].

Cefotaxime was tested in two concentrations, 40 mg/ml and 10 mg/ml, since both are frequently used in the NICU. With a battery of methods, all measurements were found to be within acceptance limits; thus, we concluded that cefotaxime is compatible with *aq*Numeta G13. This finding is in line with the conclusion of Trissel et al. who tested cefotaxime 20 mg/ml with nine different parenteral nutrition bags included in their study [30]. Cefotaxime possessed a slight, inherent Tyndall effect after reconstitution, even though the solutions were filtered 0.22 µm as part of preparation. The same weak Tyndall effect could also be seen when cefotaxime was mixed with morphine and *aq*Numeta G13E. Since cefotaxime did not reveal any signs of incompatibility with *aq*Numeta G13E in the other analyses performed in this study, it was assumed to be an effect of colour disturbance. When it comes to emulsion stability, a slightly elevated PFAT5 was found for the 4-h sample of one mixing ratio for cefotaxime with Numeta G13E + . Again, the three-component mixture did not show any increases in PFAT5, and therefore, this was not assumed to be a sign of destabilisation upon mixing.

An interesting study analysed retrospective and prospective data on drug administration of drugs and evaluated the compatibility of frequent combinations in the PICU of an Indonesian hospital [32]. Hanifah et al. explored the compatibility by looking at the single time of administration (STA) approach where bolus and intermittent drugs are given consecutively, but also together via three-way connector, through the single lumen peripheral catheter. They found that three infusions typically met sequentially and have the potential to interact. The most frequent combinations identified included some of the drugs in the current study, namely, triple combinations with morphine and dobutamine, where we studied dopamine. Moreover, Hanifah has rebuilt the infusion model with the tubing and connectors used in the clinic area in the laboratory and monitored what came out [33]. This interesting setup should be further employed.

Our results showed that the studied combinations were compatible for the specific drug products when using drug concentrations and infusion rates clinically used in the neonatal patient. It should be kept in mind that drug products from different manufacturers can have different formulations and excipients, and that both factors can influence compatibility [19]. Altogether, our results indicate that the emulsion of Numeta G13E is stable upon contact with morphine, dopamine and cefotaxime up to 4 h and no formation of precipitate should be expected; hence, co-administration of two- or three-component combinations of these drugs and PN should be safe.

Our results should be interpreted with the following limitations in mind. Only one person performed the analyses of each test set, which could, especially in the case of visual examination, have been subjective. All samples were prepared, stored and analysed at room temperature, but in the neonatal intensive care setting the drugs could be exposed to higher temperature within the neonatal ward and because of the incubators that are keeping the newborn body temperature stable. This could affect the stability of the drugs negatively, e.g. precipitation of poorly soluble calcium phosphate may increase with increased temperature [34]. Effects of incubator temperatures are not captured in the current study. The simulated Y-site compatibility analysis was performed in test tubes whereas the drugs and PN are in reality co-infused and meet in the catheter line. The liquid dynamics could introduce effects that are not accounted for in test tubes. However, since several mixing ratios were evaluated using several different analysis methods that support the same conclusions, the findings account for considerable variation and are assumed to be robust.

## Conclusions

The results of this study indicate that Numeta G13E should be compatible in co-infusion with morphine, dopamine and cefotaxime, respectively, but also in three-component infusions together with morphine + dopamine and morphine + cefotaxime. In addition, the drug + drug combinations of morphine + dopamine and morphine + cefotaxime were compatible. These findings are reassuring and contribute to safe and effective administration of drugs in the same catheter line as Numeta G13E in the neonatal intensive care patient.

## Data Availability

Not applicable.

## Abbreviations

*aq*Numeta G13E: Version of the PN product with lipid phase replaced with water
CVC: Central venous catheter
FNU: Formazine nephelometry units
MDD: Mean droplet diameter
Mw: Molecular weight
NICU: Neonatal intensive care unit
Numeta G13E +: Full version of the PN product with lipid phase and all additives
OUS: Oslo University Hospital
PDI: Polydispersity index
PFAT5: Percentage of fat residing in globules larger than 5 µm
PICC: Peripherally inserted central catheter
PN: Parenteral nutrition
SmPCs: Summary of product characteristics
